# A temperature sensor based on Si/PS/SiO_2_ photonic crystals

**DOI:** 10.1038/s41598-023-48836-5

**Published:** 2023-12-06

**Authors:** Arafa H. Aly, B. A. Mohamed, M. Al-Dossari, D. Mohamed

**Affiliations:** 1 TH-PPM Group, Physics Department, Faculty of Sciences, Beni-Suef University, Beni Suef, 62514 Egypt; 2 Department of Physics, Faculty of Science, King Khalid University, 62529 Abha, Saudi Arabia

**Keywords:** Materials science, Mathematics and computing, Nanoscience and technology, Optics and photonics, Physics

## Abstract

The present research deals with the extremely sensitive temperature-sensing capabilities of defective one-dimensional photonic crystal structures (Si/PS/SiO_2_). The proposed structure is realized by putting a defective layer of material silicon Dioxide (SiO_2_) in the middle of a structure consisting of alternating layers of silicon (Si) and porous silica (PS). The transfer matrix method has been employed to examine the transmission characteristics of the proposed defective one-dimensional photonic crystal in addition to MATLAB software. The transmission spectra of the proposed structure in the visible light domain are computed throughout a temperature range of 25–900 °C, and we study the thermal properties related to the defective mode. Additionally, the impacts of changing the defect layer's thickness are examined. Due to the effects of thermal expansion and the thermo-optical coefficient, the defect mode varies significantly as the temperature increases. Our investigation shows that the proposed structure considerably impacts the transmission intensity of the defective mode. The theoretically obtained numeric values of the quality factor and sensitivity are 2216.6 and 0.085 nm/°C, respectively. The challenges presented by conventional temperature sensors could be overcome by the suggested defective photonic crystal sensor. These results are enough to support our claim that the present design can be used as an ultra-sensitive temperature sensor.

## Introduction

Temperature sensing is likely the most crucial aspect in all research sectors. Aerodynamics, metrology, climate and marine research, medicine, chemistry, biology, and military technology are just a few industries that frequently use temperature sensors. In addition, they are used in various heating and cooling systems, food storage, and other applications^1,2^.

A lot of researchers have also looked at temperature sensors based on Z-shaped ring resonators, notably Benmerkhi et al.^3^. The study conducted by Chen et al.^4^ on the simultaneous sensing of gas concentration and temperature in one-dimensional photonic crystals. A recently developed physically meaningful model was used to study the temperature dependence of refractive indices for crystalline and amorphous silicon and to determine the indices at any operating temperature and wavelength. A temperature sensor system based on a two-dimensional (2-D) photonic crystals (PhCs) microcavity connected to two waveguides was also proposed by Ghosh^5^ and Hocini^6^.

Extensive research work on PhCs has been done so far. The present involvement of PhCs in variety of modern applications makes them suitable to be used as a promising candidate in research fields governing optical, chemical and medical engineering.

The developed periodic structures known as photonic crystals exist in one, two, and three dimensions^7–10^. As a result of this periodicity, regions known as photonic band gap regions are created, which block the propagation of light through them. Over the past few decades, a large number of scientists have become interested in these types of structures because of their ability to modify light propagation^11–14^. The one-dimensional photonic crystal is considered the easiest to produce and design out of the three kinds of photonic crystals. One-dimension photonic crystals are beneficial for switches^15^, sensors^16^, and optical filters^17–19^.

Recently, PCs have significantly improved temperature, fluid, and biological sensors^20,21^. This might be explained by the superior sensitivity, accuracy, and stability of PC-based sensors. In particular, a lot of attention has been drawn to the material stability of PC temperature sensors throughout a wide range of temperature differences and the absence of any electronic components^22,23^.

The devices' quick temperature detection, which is necessary in many different industries, allows for precise temperature measurements. Most traditional temperature sensors, such as platinum resistance thermometers, rely on resistance that change with temperature. These sensors, however, are susceptible to mechanical shock and the environment^24^. It will, therefore, quickly deteriorate over time. There are temperature sensors that use a one-dimensional photonic crystal to get past these limitations. In addition to offering excellent temperature sensitivity, this prevents costly and challenging sensor recalibration^25,26^.

In order to build an optical filter with temperature-sensitive spectrum features, most optical temperature sensors utilize the thermo-optical and physical changes that heat causes in the materials. We theoretically demonstrate how to build a temperature sensor using a defective one-dimensional photonic crystal. Temperature control of the defect peak wavelength is possible because temperature fluctuation influences the refractive index and the thickness of the defect layer. The following concept governs how a photonic crystal used as a temperature sensor works. The materials that make up a crystal will experience a change in their index of refraction as a result of the thermo-optical effect as the temperature changes around them. The thickness of the photonic crystal's layers will also alter as a result of the thermal expansion effect. In this case, the transmittance spectrum will alter. The shifts in the transmittance spectrum can be used to detect a little change in temperature. One approach to effectively utilizing photonic crystals as temperature sensors is demonstrated here^27^. Due to the greatly improved performance of such structures in terms of sensitivity (S), figure-of-merit (FoM), quality factor (Q), and detection limit (DL), photonic biosensors composed of 1D DPhCs have changed the planning, development, and testing methods of biosensors. In order to achieve high-field localization, 1D DPhCs-based photonic biosensors really enhance the interaction time between incident photons and the target matter procured inside the sensing zone of the structure. While the fascinating properties of 1D DPhCs biosensors, such as tunable dispersion and birefringence, homogeneous and isotropic behavior of the structure, and device compatibility, facilitate the fabrication of 2D DPhCs biosensors, their fabrication is restricted by interference, phase, and group birefringence. Depending on the material chosen for this type of design, it is possible to produce these structures in a simple manner by applying appropriate thin film deposition techniques. The architectural details, along with the theoretical formulation of the structure, are discussed in Section “Basic equations and model design”. The results of the work are presented in Section “Results and discussions”. Finally, conclusions are summarized in Section “Conclusion”.

## Basic equations and model design

**Figure 1:**
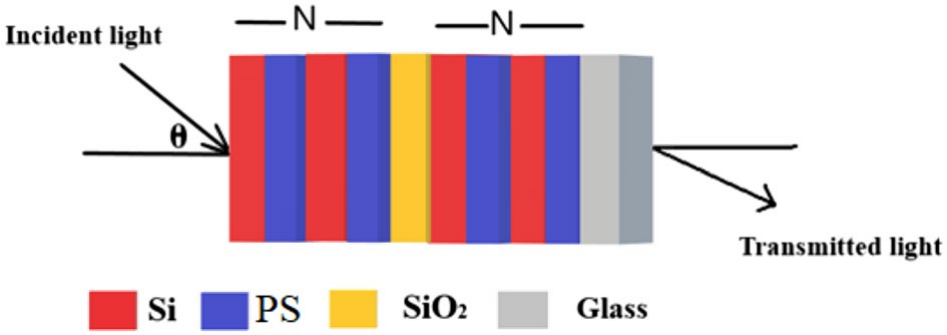
a systematic view of the proposed 1D DPhC composed of Si/PS/SiO_2_/Glass. The red, blue, yellow red and grey colours are representing different layers of the structure made up of Si, PS, SiO_2_ and glass materials respectively.

The transfer matrix method (TMM) has been employed to investigate the theoritical findings of the 1D DPhC composed of (Si/PS/SiO_2_) as shown in Fig. 1. The present structure can be fabricated by depositing the alternating periodic layers of silicon (Si) and fused silica (SiO_2_) of period number *N* on either side of an additional defect layer of Silicon Dioxide (SiO_2_) located at the middle of the structure over glass substrate.

A one-dimensional photonic crystal structure with N periods of Si and PS defective with SiO_2_ layers, whose thicknesses are d_1_, d_2_, and d_d_ correspondingly, makes up the suggested sensor's schematic representation in Fig. 1. The thicknesses of Si = 44 nm, P = 103 nm, SiO_2_ = 100 nm as well as N = 5 periods with normal incidence.

We have considered T = 25 °C as room temperature which will be the temperature reference here. Additionally, due to the thermal-expansion effect and thermo-optical effect, the refractive indices and thicknesses of these layers can be represented as a function of temperature^28^. We may express the changes in constituent material thicknesses caused by temperature as, $d={d}_{o}+\alpha {d}_{o}\Delta T$, where d_0_ is the thickness at room temperature and $\alpha$ is the thermal expansion coefficient. According to the thermo-optical effect, the refractive indices depend on the temperature as follows: $n={n}_{o}+\beta {n}_{o}\Delta T$, where n_o_ is the refractive index at room temperature and $\beta$ is the thermo-optic coefficient. Equation (1) below defines the transfer matrix technique, which connects the electric and magnetic fields of electromagnetic waves flowing into the structure close to the interface between every two adjacent layers of the structure^9,29–32^.

1 $$ {\text{M}} = \left[ {\begin{array}{*{20}c} {{\text{M}}_{11} } & {{\text{M}}_{12} } \\ {{\text{M}}_{21} } & {{\text{M}}_{22} } \\ \end{array} } \right] = \left( {{\mathfrak{M}}_{1} {\mathfrak{M}}_{2} } \right)^{N} {\mathfrak{M}}_{d} \left( {{\mathfrak{M}}_{1} {\mathfrak{M}}_{2} } \right)^{N} $$

Here M_11_, M_12_, M_21_ and M_22_ are representing the elements of the resultant transfer matrix M of the structure. The symbols $\mathfrak{M}$_1_, $\mathfrak{M}$_2_ and $\mathfrak{M}$_d_ are representing the characteristic matrix of material layers Si, PS and SiO_2_ of the structure. The subscripts 1, 2 and d are being used material layers Si,PS, and SiO_2_ respectively of the structure. The characteristic matrix $\mathfrak{M}$_n_ (*n* = 1, 2 and d) of any n-th layer of the structure can be defined as

2 $$ {\mathfrak{M}}{\text{n}} = \left[ {\begin{array}{*{20}c} {\cos \left( {k_{nz} d_{n} } \right)} & {jq_{l}^{ - 1} \sin \left( {k_{nz} d_{n} } \right)} \\ {jq_{n} \sin \left( {k_{nz} d_{n} } \right)} & {\cos \left( {k_{nz} d_{n} } \right)} \\ \end{array} } \right] $$

The reflection (r) and transmission (t) coefficients of the proposed structure (Si/PS/Si/SiO_2_) are defined by

3 $$ r = \frac{{M_{21} }}{{M_{11} }},\quad t = \frac{1}{{M_{11} }} $$

The reflectance (*R*) and transmittance (*T*) of the structure can be with the help of following relation (4)

4 $$ R = \left| r \right|^{2} = \left| {\frac{{M_{21} }}{{M_{11} }}} \right|^{2} ,\quad T = \frac{{p_{s} }}{{p_{0} }} \left| t \right|^{2} = \frac{{p_{s} }}{{p_{0} }} \left| {\frac{1}{{M_{11} }} } \right|^{2} $$

Here p_0_ = n_0_ cos(θ_0_) and p_s_ = n_s_ cos(θ_s_) are admittances of input and output ends of the structure respectively, corresponding to s-polarized wave. For p-polarized wave, input and output admittances would be p_0_ = cos(θ_0_)/n_0_ and p_s_ = cos(θ*s*)/n_s_ respectively. The symbols θ_0_ and θ_s_ are denoting angles of incidence and emergence at input and output ends of the structure respectively.

The sensitivity of the photonic crystal temperature sensor is one of many parameters that may be used to assess its performance. Which is the rate of change in the position of the defect mode, $\Delta \lambda ,$ to the change in temperature of the defect layer, ΔT, where we use here the sensitivity; S = Δλ/ΔT nm/°C.

The figure of merit (FOM) is a numerical amount that reflects a measure of efficiency or effectiveness based on one or more attributes of a system or device, which determines any little change in the position of the defect peak inside PBG and it is affected by both sensitivity and the bandwidth (FWHM) of resonant peak, as seen follow;$FOM=S/FWHM$, Quality factor is very important parameter in any sensor device which determined by simple equation; $Q=\frac{{\lambda }_{d}}{FWHM}$

### Consent to participate

All authors accepted.

## Results and discussions

In this section, the findings will be simulated, and the suggested sensor will be thoroughly covered. The proposed structure (1D-DPhC) is meant to transmit, absorb, and reflect light in a manner that enables it to sense biological molecules or other chemicals. The proposed structure's interaction with light is what drives the recommended sensor. A defective layer of silica (SiO_2_) is sandwiched between the two components in this structure, which are silicon (Si) and porous silica (PS), both of which are repetitive and have distinct refractive indices, and this structure is surrounded by air and substrate of glass as incidence and output media respectively so that the final configuration of our structure becomes as air/(Si/PS)^N^/SiO_2_/(Si/PS)^N^/substrate. Table 1 lists the refractive indices, thickness (nm), thermal, optical coefficient (°C^−1^), and thermal expansion (°C^−1^) of three samples at room temperature.

**Table 1:** Thermal parameters of the chosen material at room temperature ^29,33^.

Material	Refractive index	Thickness (nm)	Thermal optical coefficient (°C^−1^)	Thermal expansion (°C^−1^)
Silicon (Si)	3.49	44	1.86 × 10^−4^	5 × 10^−7^
Silica (SiO_2_)	1.45	100	1 × 10^−5^	5.5 × 10^−7^
Porous Silica (PS)	1.5916	103	1.2 × 10^–4^	2.2 × 10^–6^

**Figure 2:**
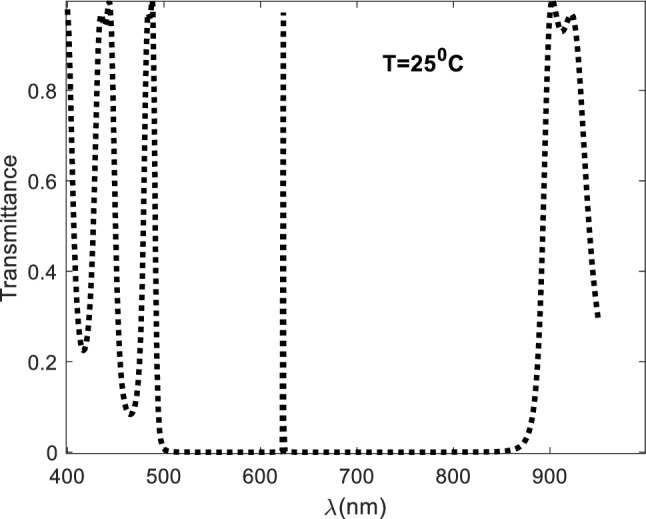
Transmittance spectra for 1D defected photonic crystal (Si/PS)^N^ SiO_2_(Si/PS)^N^ at room temperature.

Based on the aforementioned values, Fig. 2 plots the structure's corresponding transmittance spectrum. According to Fig. 2, the photonic band gap has a resonant peak at a wavelength region of 623.7 nm, a significant forbidden region in the wavelength range of 488.9115: 899.5978 nm. Certain wavelengths of incident electromagnetic waves are prevented from passing through the structure at this range. The large difference in refractive indices between silicon (Si) and porous silica (PS) causes the photonic band gap to be so wide.

**Figure 3:**
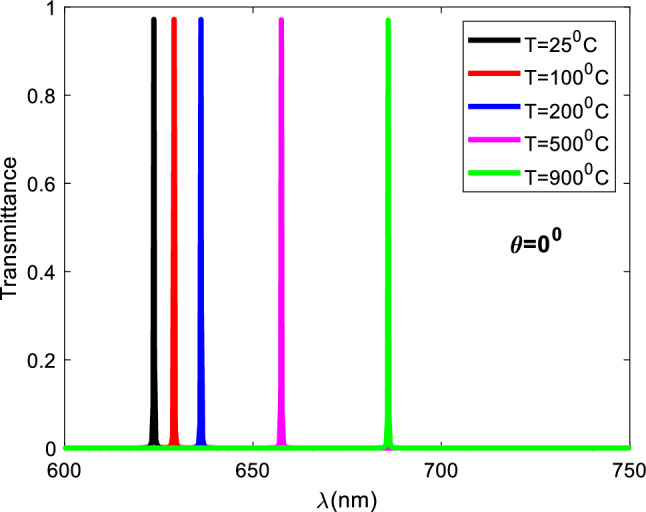
Resonant peaks for defective layers at different temperatures, normal incidence, and number of periods N = 5.

The refractive indices of the several defective layers employed in this study can be determined using the following relationship. $n={n}_{0}+\beta {n}_{0}\Delta T$, Hence, when all other factors are constant, the refractive index of the defective layer fluctuates with temperature. According to Fig. 3, the defect layer's refractive index increases as the temperature increases, and the resonant peak shifts toward the higher wavelength area. Table 2 lists all of the defective layers' refractive index values employed at various temperatures, along with the location of each refractive index's corresponding resonant peaks.

**Table 2:** Relation between refractive index of defect layer (RIU), peak wavelength (nm), and temperature(c).

Refractive index of defect layer (RIU)	Temperature (°C)	Peak wavelength (nm)
1.45	25	623.7
1.451	100	629.1
1.452	200	636.2
1.456	500	657.5
1.462	900	685.9

**Figure 4:**
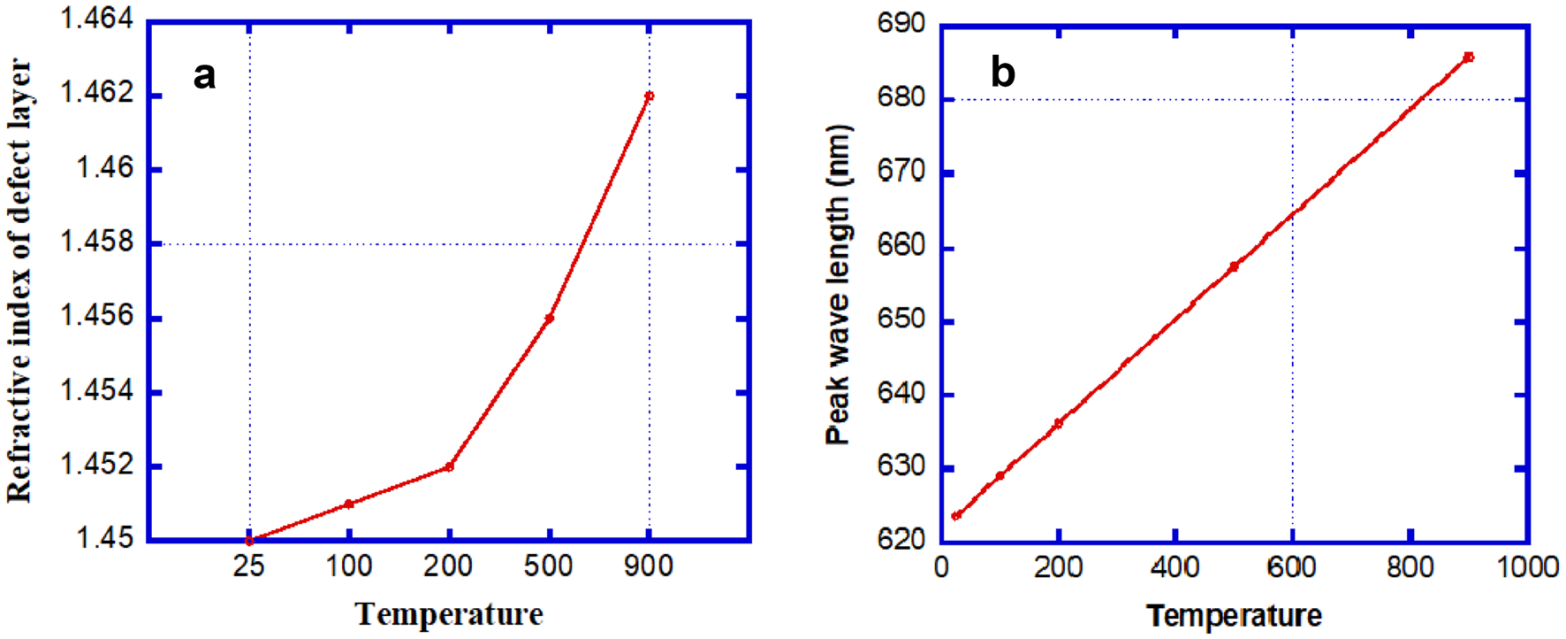
Diagrams illustrate the changing of refractive index of defect layer (RIU) (**a**) and peak wavelength (nm) (**b**) with the change of temperature respectively at normal incidence, and number of periods N = 5.

In Fig. 4a,b, there is a direct relationship between the temperature and the refractive index of the defective layer as well as between the temperature and the change in wavelength position, respectively. In materials such as organic silica SiO_2_, the increase of the refractive index of the defect layer with the increase of temperature can be explained by the following direct relation: $n={n}_{o}+\beta {n}_{o}\Delta T$ where the refractive index at room temperature (no) and the thermo-optic coefficient ($\beta )$ are constant.

### The effect of the thickness of the defect layer on sensitivity and resonance location

The sensitivity improved as a result of the inquiry into changing the thickness of the fault layer. The change was studied using three unique thicknesses of 100, 150, and 200 (nm), and the sensitivity values for each thickness were 0.072, 0.073, and 0.085 (nm/°C), respectively. With increasing defect layer thickness, the structure absorbs and emits more light, which improves the interaction between light and the defect layer in the crystal. The thickness of 200 nm, which is regarded as the optimal thickness, allowed for achieving the maximal sensitivity for a typical incidence.

**Figure 5:**
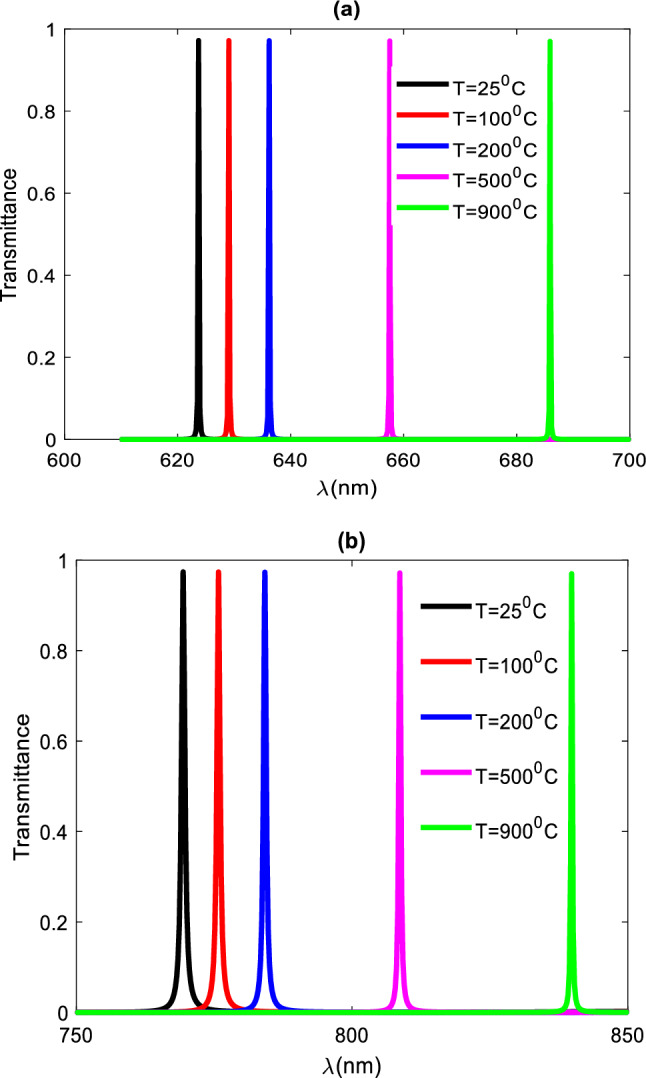
(**a**) Transmittance spectra at dd = 100 nm the thickness of defect layer, (**b**) is the transmittance spectra at dd = 200 nm at normal incidence and N = 5 period.

Figure 5a,b displays a shift in the defect peak positions for the same defect layers at the same temperatures but using different 100 and 200 nm thicknesses, respectively.

**Figure 6:**
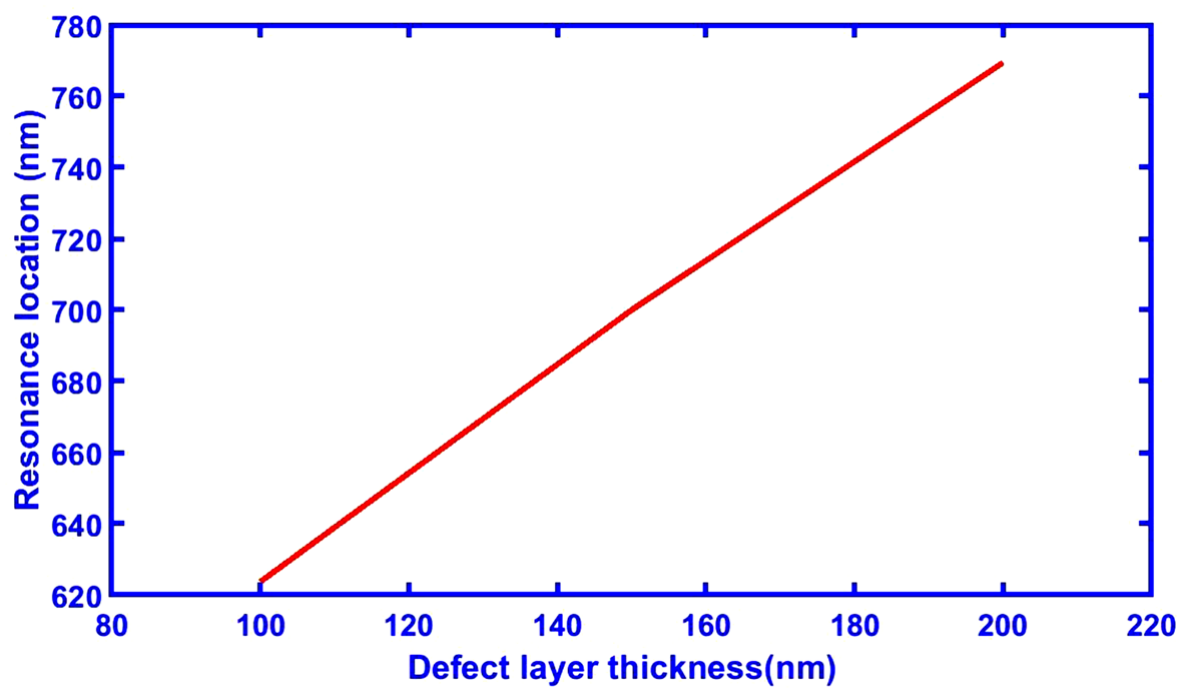
A direct relation showing the changing of defect layer thickness and its corresponding resonance locations at normal incidence, and number of periods N = 5.

Figure 6 explains the connection between the thickness of the defective layer and the resonance position, and Table 3 provides all the sensitivity and resonance position values that correspond to each thickness. The defect position shifts to a higher region in the wavelength as the thickness of the defective layer increases.

**Table 3:** Values of sensitivity and resonance position at different thickness of defect layer.

Defect layer thickness (nm)	Sensitivity nm/°C	Resonance location (nm)
100	0.072	623.7
150	0.073	700
200	0.085	769.4

### The effect of incident angle on sensitivity

**Figure 7:**
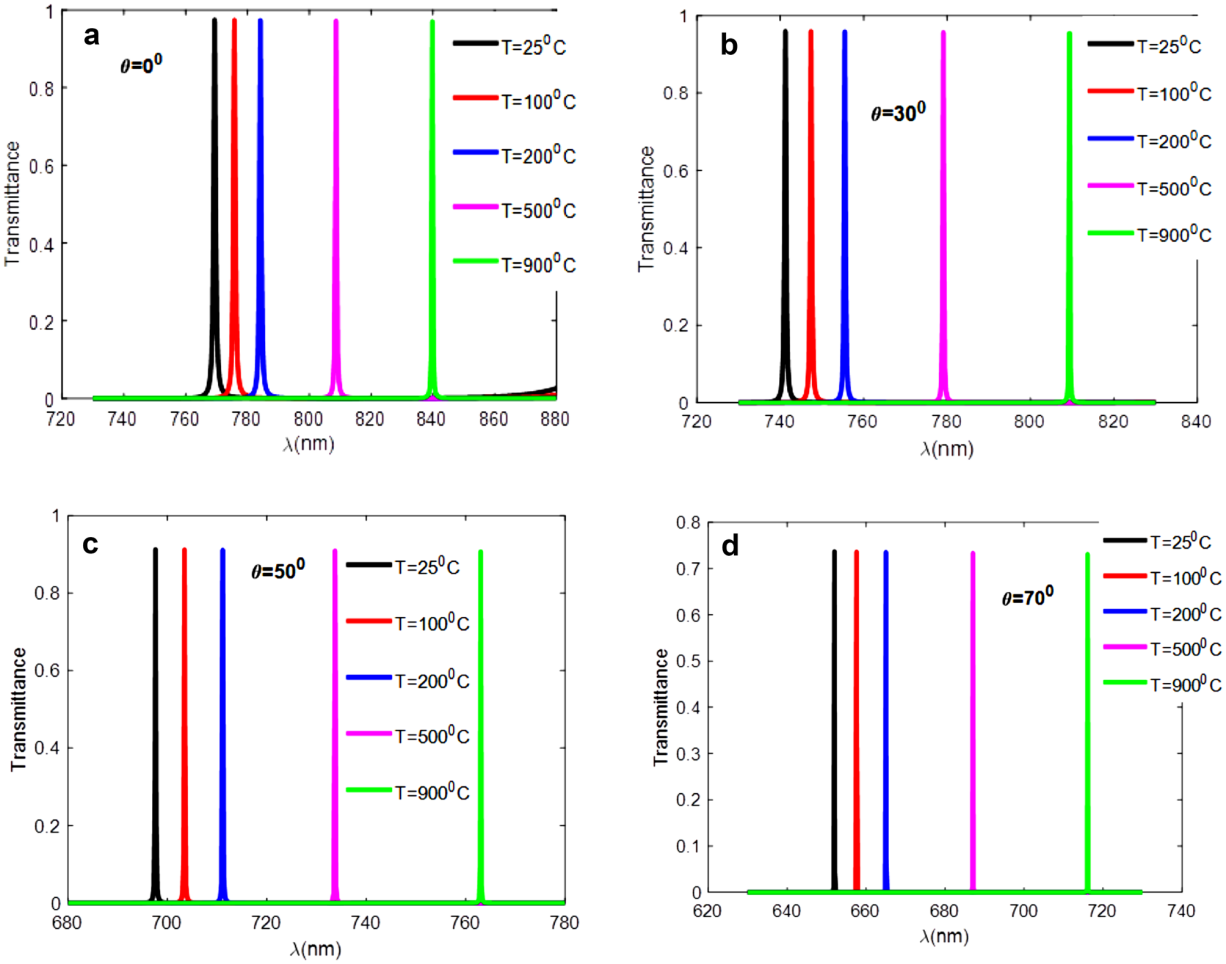
Peak positions of 1D-DPhC with optimum defective layer thickness dd = 200 (nm) when the angle of light incidence (**a**) θ = 0°, (**b**) 30°, (**c**) 50°, and (**d**) 70° respectively.

This study examined the effect of the incidence angle at the optimal thickness of 200 nm for defect layers and observed how its modification affected the suggested structure's sensitivity. According to Fig. 7, this shift was examined at angles of 0°, 30°, 50°, and 70°, respectively. We notice from Fig. 7 that the transmittance decreases with the increase in the angle of incidence from 0° to 70° whereas the ratio of reflectance of the incident light increases. It is worth noting that absorption here is a very small amount, approximately 6.608 x 10^–13^ at room temperature, and therefore does not affect our results, so it cannot be considered. According to the study's findings, the sensitivity of the structure decreased from 0.085 to 0.074 (nm/°C) when the angle of incidence increased from 0° to 70°, with the normal incident angle yielding the highest sensitivity value. Table 4 contains a list of all the selected angles together with the sensitivity levels for each angle. For further clarification in the defective layer, we plotted the transmittance spectrum at different incidence angles of TE polarization.

**Table 4:** Values of Incident angles and corresponding sensitivity values at optimum defective layer thickness dd = 200 (nm).

Incident angle (°)	Sensitivity (nm/°C)
0	0.085
30	0.082
50	0.0773
70	0.074

The effect of effective parameters and other factors, such as ${\lambda }_{FWHM}$, quality factor and FOM of the proposed sensor have been examined in Table 5 below; depending on the change in temperature from Table 5 data, we can see that when the temperature rises from 25 to 900 °C, that ${\lambda }_{FWHM}$ reduces from 0.4 to 0.1 nm. Additionally, the quality factor rises from 1923.5 to 8398 at the same range of temperatures, and the FOM rises with increasing temperature until it reaches its highest value, which is 0.8 at a temperature of 900 °C. According to the specified temperature, Table 5 contains all values for the refractive index, resonance peak, sensitivity,${\lambda }_{FWHM}$, Q-factor, and FOM.

**Table 5:** Theoretical calculations showing numerical values of refractive index, resonance peak, sensitivity, ${\uplambda }_{\mathrm{FWHM}}$, Q- factor and FOM due to change in the temperature of defect layer sample at dd = 200 nm, when the angle of light incidence θ = 0° and N = 5 period.

Temperature (°C)	Refractive index (RIU)	Resonance peak (nm)	Sensitivity (nm/°C)	${\lambda }_{FWHM}$ (nm)	Q- factor	FOM
25	1.45	769.4	–	0.4	1923.5	–
100	1.451	775.8	0.085	0.35	2216.6	0.0243
200	1.452	784.2	0.084	0.3	2614	0.28
500	1.456	808.7	0.083	0.2	4043.5	0.42
900	1.462	839.8	0.0804	0.1	8398	0.8

The results obtained for our suggested structure in terms of sensitivity are compared with some earlier published results as well as comparing the outcomes of the proposed sensor to those of previous studies^26–28^; the best sensitivity was found to be 0.085 nm/°C as shown in Table 6.

**Table 6:** Comparison depending upon temperature sensitivity between our proposed results with the previous works.

Author and Title	Year	Sensitivity (nm/°C)
Juan Zhang et al., “Enhanced temperature sensing based on sub-threshold nonlinear spectra of one-dimensional photonic crystal with a Kerr defect layer”^34^	2014	0.0049
Yang-Hua Chang et al., “Temperature dependence of defect mode in a defective photonic crystal”^21^	2012	0.0037
Kumar, A, et al. , “Wide range temperature sensors based on one dimensional photonic crystal with a single defect”^35^	2012	0.064
Proposed work	2023	0.085

## Conclusion

In conclusion, we used the transfer matrix approach to investigate the thermal characteristics of our suggested construction. Our structure is made up of a SiO_2_ layer in the middle of a straightforward one-dimensional photonic crystal defect. Temperature-dependent transmittance spectra in the visible light spectrum were investigated. For various temperatures, the impact of adjusting the thickness of the defect layer and incident angle is also investigated. Our findings indicate that the suggested structure has a considerable impact on the transmission intensity of the defective mode. The sensitivity for this work is 0.085 nm/°C, the figure of merit is 0.0243, and the high-quality factor is 2216.6. Conventional temperature sensors have challenges such as difficulty in fabrication, high cost and low performance, and these sensors are extremely susceptible to mechanical shock and the environment; as a result, they will quickly deteriorate over time that the suggested defective photonic crystal sensor could address. The suggested sensor might be helpful in the creation of a cheap and practical temperature sensor.

## Data Availability

The data that support the findings of this study are available from the corresponding author upon reason-able request.
